# Serial analysis of gene expression reveals differential expression between endometriosis and normal endometrium. Possible roles for AXL and SHC1 in the pathogenesis of endometriosis

**DOI:** 10.1186/1477-7827-6-59

**Published:** 2008-12-02

**Authors:** Hiroshi Honda, Fermin F Barrueto, Jean Gogusev, Dwight D Im, Patrice J Morin

**Affiliations:** 1 Laboratory of Cellular and Molecular Biology, National Institute on Aging, Baltimore MD 21224, USA; 2 The Gynecology Center, Mercy Hospital, Baltimore, MD 21202, USA; 3 INSERM U507, Hôpital Necker, Paris, France; 4 Department of Pathology, Johns Hopkins Medical Institutions, Baltimore, MD 21287, USA

## Abstract

**Background:**

Endometriosis is a clinical condition that affects up to 10% of the women of reproductive age. Endometriosis is characterized by the presence of endometrial tissues outside the uterine cavity and can lead to chronic pelvic pain, infertility and, in some cases, to ovarian cancer.

**Methods:**

In order to better understand the pathogenesis of endometriosis, we have used Serial Analysis of Gene Expression (SAGE) to identify genes differentially in this disease by studying three endometriotic tissues and a normal endometrium sample. Promising candidates (AXL, SHC1, ACTN4, PI3KCA, p-AKT, p-mTOR, and p-ERK) were independently validated by immunohistochemistry in additional normal and endometriotic tissues.

**Results:**

We identified several genes differentially expressed between endometriosis and normal endometrium. IGF2, ACTN4, AXL, and SHC1 were among the most upregulated genes. Comparison of the endometriosis gene expression profiles with the gene expression patterns observed in normal human tissues allowed the identification of endometriosis-specific genes, which included several members of the MMP family (MMP1,2,3,10,11,14). Immunohistochemical analysis of several candidates confirmed the SAGE findings, and suggested the involvement of the PI3K-Akt and MAPK signaling pathways in endometriosis.

**Conclusion:**

In human endometriosis, the PI3K-Akt and MAPK signaling pathways may be activated via overexpression of AXL and SHC1, respectively. These genes, as well as others identified as differentially expressed in this study, may be useful for the development of novel strategies for the detection and/or therapy of endometriosis.

## Background

Endometriosis is a common gynecological disorder that affects 5–10% of women of reproductive age and that causes dysmenorrhea, chronic pelvic pain, and infertility [1]. Despite numerous studies on endometriosis, its etiology and pathogenesis have not yet been fully elucidated. The disease is characterized by the presence of endometrial epithelial and stromal cells outside the uterine cavity. Biologically, these ectopic cells are quite distinct from their eutopic counterparts. Different responses to steroid hormones, growth factors and cytokines, chromosomal aberrations [2-4], Loss of heterozygosity (LOH), or allelic loss of specific chromosomal regions [5-8] have been reported. Furthermore, especially in the case of ovarian endometriosis, malignant transformation to ovarian cancer has been suggested [9,10]. On the basis of these findings, ovarian endometriosis has been considered to be a precursor lesion of ovarian carcinoma [11,12].

In the past several years, a number of microarray studies have been performed on endometriosis tissues [13-26]. Although these studies have identified large numbers of genes differentially expressed between endometriotic tissues and eutopic endometria, few studies could identify specific molecular pathways that are commonly involved in the pathogenesis of this disease.

Serial analysis of gene expression (SAGE) is a high-throughput gene expression profiling approach that can be considered complementary to microarray approaches [27-29]. Although SAGE is labor intensive, there are some advantages in SAGE compared to microarray. SAGE is an open system and does not require prior knowledge of the genes of interest, allowing the identification of novel genes. In addition, because SAGE is a sequencing-based approach, this method does not have the well known limitations inherent to hybridization-based assays such as differences in hybridization capabilities of various targets and normalization issues. Furthermore, because the gene expression quantitation is absolute rather than relative, data obtained by SAGE can be compared with experiments from different laboratories done at different times [30]. However, because of the labor required in the preparation of the libraries, as well as the high cost of sequencing them (cost may be 5–10 times higher than microarray), the number of samples analyzed is typically low compared to microarray studies, and validation of the candidates in additional samples is crucial [28,29]. Because of the differences between SAGE and microarray approaches (i.e., sequencing vs hybrization), each technique may be better than the other at identifying certain genes, and indeed, previous SAGE studies have been instrumental in identifying candidate genes that had not been found using microarray techniques [31-34]

Because of its possible role as a precursor of ovarian cancer, we focused this study on ovarian endometriosis. We performed large-scale gene expression profiling of these lesions using SAGE and compared the gene expression profiles with those of eutopic normal endometrium. Because of their expression pattern, *SHC1*, *ACTN4*, and *AXL*, were identified as promising endometriosis-related candidate genes, and their expression patterns were further investigated at the protein level by immunohistochemistry.

## Methods

### Patient Samples for SAGE

Two ovarian cysts of r-AFS (revised American Fertility Society Classification for Endometriosis) stage IV endometriosis and one normal eutopic endometrium without endometriotic lesion (control) were obtained anonymously from premenopausal women during mid to late proliferative menstrual phase at Mercy Hospital (Baltimore, MD, USA). The control patient was confirmed to have no endometriotic or other pathological lesions in the pelvic cavity. These patients did not take any hormonal therapy prior to their surgery. The patients gave informed consents and the present study was approved by the NIH Institutional Review Board and by the ethics committee of the Mercy Hospital, Baltimore, MD, USA. Endometriotic tissue layers were scraped from the inner wall of the cyst, minced into small pieces, and enzymatically dissociated by incubation with 0.25% collagenase (Sigma, St. Louis, MO, USA) and 0.02% DNase I in phenol-red-free Dulbecco's modified Eagle's Medium/Ham's F-12 (Invitrogen, Carlsbad, CA, USA) supplemented with 10% FBS (Invitrogen) for 1 hr at 37°C. Enrichment of the endometriotic epithelial cells was performed by serial filtration using 100 μm and 40 μm nylon sieves (BD Falcon, Franklin Lakes, NJ, USA), and collected by back-washing the 40 μm nylon sieve onto tissue culture dishes. After incubation at 37°C for 30 minutes to allow stromal cells to attach to the dishes, the floating endometriotic epithelial cells in the supernatant were transferred into a T25-culture flask and cultivated for approximately 7 days. The endometriotic epithelial cells were harvested at 80% confluence, resuspended in the Lysis/Binding Buffer of the Dynabeads^® ^mRNA DIRECT™ Kit (Invitrogen), immediately snap-frozen and kept at -80°C until further processing. The purity of the epithelial cells was assessed by immunopositivity of cytokeratin. A similar procedure was used for the isolation of eutopic endometrial epithelial cells, except that the isolation procedure started with the mincing of the normal endometrium. Approximately, 5.0 × 10^5 ^to 1.0 × 10^6 ^cells were lysed in each sample. An endometriotic cell line, FbEM-1, which was previously established from ovarian and peritoneal endometriosis [35] was also used for the present study. Total RNA of the FbEM-1 cell line was extracted and used for SAGE.

### SAGE

Each SAGE library was made according to the microSAGE protocol with minor modifications. Polyadenylated mRNA was purified on Oligo (dT)_25 _from the cell lysates by using the Dynabeads^® ^mRNA DIRECT™ Kit (Invitrogen) for the short-term cultivated cells, and from 10 μg of total RNA by using the Dynabeads^® ^mRNA Purification Kit for the FbEM-1 cell line. cDNA was generated using the SuperScript™ Double-Stranded cDNA Synthesis Kit (Invitrogen). The rest of the SAGE procedure was performed essentially as described [36]. Colonies on the agar lawns were picked and sequenced by Agencourt (Boston, MA).

### SAGE Data analysis

Sequence data from each library were analyzed by the SAGE software SAGE2002 to quantify tags. Tag numbers were normalized to 200,000 to allow for direct comparisons of the different libraries. For further analysis, the SAGE data was imported from the SAGE2002 software into excel (Microsoft) spreadsheets. For the identification of endometriosis-specific transcripts data was downloaded from the SAGE Genie database  into excel and compared to the endometriosis data obtained in this study. Gene expression data was obtained for the following 29 normal tissues: ovarian surface epithelial cells, endometrium, peritoneum, colon (2 libraries), prostate (2 libraries), pancreas (2 libraries), skin, thyroid, white blood cells, bone marrow, brain cortex, thalamus, cerebellum, stomach (2 libraries), heart, kidney, liver, lung, lymph node, breast myocytes, breast stroma cells, breast epithelial cells, muscle (2 libraries), and vascular endothelial cells. Tags expressed at a level of more than 20 tags/200,000 in endometriosis and with more than 75% of their total expression found in this tissue compared with all the other normal tissues were considered endometriosis specific. In addition, tags that were found in more than 8 of the 29 normal tissues were also eliminated from the list for lack of specificity. The 20 most highly expressed genes that satisfy those criteria were considered the best endometriosis-specific genes. Significance and *p*-values were determined using previously described approaches [37]. A p-value of < 0.05 was accepted as statistically significant.

### Immunohistochemistry (IHC)

Eight ovarian endometriosis samples from patients with r-AFS stage III-IV endometriosis without hormonal therapy (age 23–52, average 41) and 5 normal endometrium specimens from premenopausal women without endometriosis or hormonal therapy (age 36–49, average 42) were evaluated by IHC for the validation of SAGE data. The control premenauposal women underwent hysterectomy without prior hormonal therapy and were confirmed to be free of endometriosis in the pelvic cavity and the surgically resected tissues. Among 8 cases of ovarian endometriosis, three cases were in the proliferative phase of the menstrual cycle and 2 cases were in the secretory phase. No information about the menstrual phase was available for the remaining 3 cases of ovarian endometriomas. Among 5 cases of normal endometrium, three cases were in the proliferative phase of the menstrual cycle and 2 cases were in the secretory phase, respectively. Briefly, deparafinized 5-μm sections of formalin-fixed specimens of ovarian endometriosis and normal endometrium were submitted to heat-induced antigen retrieval, incubated with the specific antibodies, and processed using the Vectastain Elite ABC Kit (Vector Laboratories, Burlingame, CA, USA) with 3,3'-diaminobenzidine as the chromatogen. The sections were then counterstained with hematoxylin and mounted with coverslips. The following primary antibodies were used: AXL (polyclonal, dilution 1:50, Santa Cruz Biotech., Santa Cruz, CA, USA), SHC1 (polyclonal, dilution 1:300, Santa Cruz Biotech.), phospho-Erk1/2 (monoclonal, dilution 1:50 Santa Cruz Biotech.), PI3KCA (polyclonal, dilution 1:50, Abgent, San Diego, CA, USA), phospho-Akt (Ser473) (clone 736E11, dilution 1:50, Cell Signaling Technology, Danvers, MA, USA), phospho-mTOR (Ser 2448) (clone 49F9, dilution 1:50, Cell Signaling Technology). The antibody for ACTN4 was kindly provided by Dr. Kazufumi Honda and Dr. Tesshi Yamada (National Cancer Center Research Institute, Tokyo, Japan). Staining intensity was scored as follows: 0, none of the cells stained positively; 1, weak staining; 2, moderate staining; and 3, strong staining. Statistical analysis was done with Student's t-test to compare the staining intensity for each pair of normal endometrium and endometriotic tissue samples.

## Results

### SAGE library construction for normal and endometriotic tissues

In order to characterize the gene expression patterns associated with endometriosis, we performed SAGE on normal purified endometrial cells, two purified endometriosis tissues, and one endometriosis cell line. To ensure the integrity of the gene expression data, a high emphasis was placed on obtaining pure cell populations for our analysis. The epithelial component of the endometriotic tissues was enriched *in vitro *and the purity of the resulting cell population was confirmed by cytokeratin staining before performing SAGE. A similar approach was used in the enrichment of normal endometrial cells.

**Figure 1:**
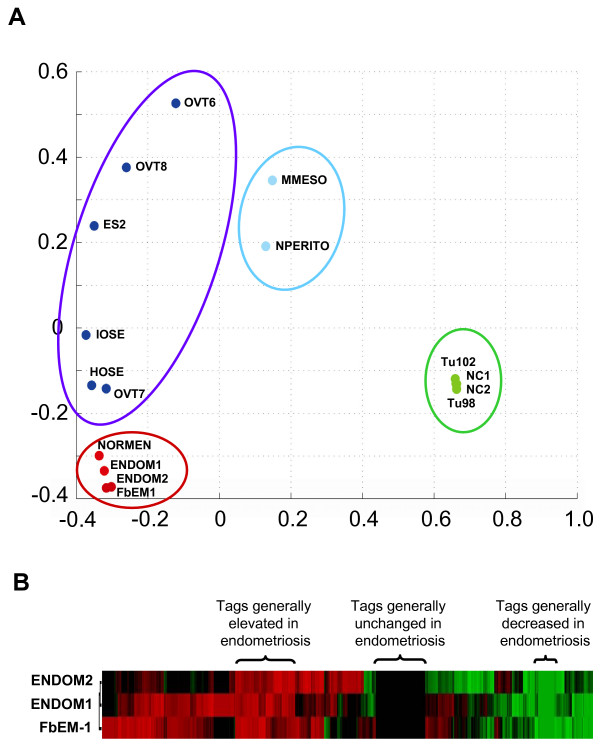
**Serial Analysis of gene expression of endometriosis**. A. 2D MDS plot shows that tissues of related origin cluster together. Endometrium and endometriosis libraries (NORMEN, ENDOM1, ENDOM2 and FbEM-1) are indicated in red, ovarian tissues (normal ovarian surface epithelial cell lines: IOSE and HOSE, ovarian cancer tissues: ES2, OVT6, OVT7, and OVT8) in dark blue, normal peritoneum (NPERITO) and malignant mesothelioma (MMESO) in light blue, and colon tissues (normal colon epithelium: NC1 and NC2, colon cancer tissues: Tu98 and Tu 102) in green. B. Clustering analysis of tags level in the three endometriosis libraries (endom1, endom2 and FbEM-1). The levels are relative to the expression in normal endometrium. Areas consisting of tags consistently elevated, decreased, or unchanged are shown above the heat map.

The construction of SAGE libraries from the 2 endometriotic tissues led to high quality libraries, endom1 and endom2, each yielding approximately 80,000 tags (Table 1). The library prepared from the endometriotic cell line (FbEM-1) also yielded a high quality library (76,100 tags). The library obtained from the purified normal endometrial cells, normen2, yielded fewer tags, but was still considered of acceptable quality with 19,493 tags. Multi-dimensional scaling (MDS) analysis showed that overall, the gene expression profiles were extremely similar between the various endometriosis tissues (and normal endometrium) when compared with other tissues included as controls, such as colon, ovarian, and peritoneal tissues (Fig. 1A).

**Table 1:** SAGE libraries information

Library name	Sequences	Total tags	Total genes	>1*
Normal endometrium	2016	19493	10089	2193
endom1	2688	80783	20727	7200
endom2	2784	77846	24329	7394
FbEM-1	2688	76100	25652	7274

*number of tags found more than once.

### Gene expression profiles in endometriosis

Cluster analysis was performed on the SAGE libraries and differentially expressed genes were identified (Fig. 1B). Table 2 shows the top 20 upregulated and downregulated genes in endometriosis compared to normal endometrial cells. Overall, a much larger number of genes were highly upregulated as opposed to downregulated. For example, we found 53 genes that were elevated more than 100-fold, but only 2 genes downregulated by at least 100-fold. Among the highly elevated genes, we found several involved in signaling such as *IGF2*, *ACTN4*, *AXL*, and *SHC1*. The list of downregulated genes (Table 2) contained several myosin and actin related genes (*MYL6*, *ACTG1*, *MRLC2*).

**Table 2:** Genes differentially expressed in endometriosis

Tag_Sequence	symbol	gene name	endom1	endom2	FbEM-1	normen2	Fold
**Elevated**							
CCAGTGGCCC	*RPS9*	Ribosomal protein S9	64	77	154	0	590
CGAGGGGCCA	*ACTN4*	Actinin, alpha 4	108	50	98	0	512
CTTGGGTTTT	*IGF2*	Insulin-like growth factor 2 (somatomedin A)	28	193	16	0	474
GTATGGGCCC	*CHI3L1*	Chitinase 3-like 1 (cartilage glycoprotein-39)	22	160	10	0	384
GCTGAACGCG	*CEBPB*	CCAAT/enhancer binding protein (C/EBP)	29	100	15	0	288
CGCAGTGTCC	*ATP6V0C*	ATPase, H+ transporting, lysosomal 16kDa	53	41	31	0	250
TGAAGTTATA	*ITGB1*	Integrin, beta 1 (fibronectin receptor)	39	37	48	0	248
AGCTACCGGG	*EFEMP2*	EGF-containing fibulin-like ECM protein 2	55	11	54	0	240
CGGCTGGTGA	*PSMB1*	Proteasome (prosome, macropain)	32	32	52	0	232
GTGCCCTGTT	*NCKAP1*	NCK-associated protein 1	42	46	20	0	216
TTGCCCCCGT	*AXL*	AXL receptor tyrosine kinase	42	51	14	0	214
GCAGTCGCTT	*DYNLRB1*	Dynein, light chain	40	37	28	0	210
CAGCTGGGGC	*PTBP1*	Polypyrimidine tract binding protein 1	27	26	52	0	210
GCCACAGTAC	*DKFZP586H2123*	Regeneration associated muscle protease	42	42	16	0	200
GCCCCGAGCC	*REEP5*	Receptor accessory protein 5	18	34	43	0	190
ACCCCCCCGC	*JUND*	Jun D proto-oncogene	35	21	38	0	188
GAAACAAGAT	*PGK1*	Phosphoglycerate kinase 1	19	28	44	0	182
GAGGGGAAAC	*SHC1*	SHC (Src homology 2 domain containing)	40	32	15	0	174
GCCAACAACG	*NNMT*	Nicotinamide N-methyltransferase	28	15	42	0	170
TTGCCCAGCA	*CEECAM1*	Cerebral endoth. cell adhesion molecule 1	33	32	17	0	164
**Decreased**							
GGGCGGAGCT	*SMARCC2*	Myosin, light chain 6	0	0	0	68	136
TAATTTTGAA	*SNHG5*	Small nucleolar RNA host gene 5	0	0	0	50	100
TCCCTATGCT	*Hs.689482*	CDNA FLJ45874	0	0	0	40	80.0
AAGTGTGACG	*ACTG1*	actin, gamma 1	0	0	0	36	72.0
AAATTTTAAA	*HP1BP3*	Heterochromatin protein 1, binding prot 3	0	0	0	31	62.0
TGGTGTTTGG	*PPIE*	Peptidylprolyl isomerase E	0	0	0	27	54.0
GGGGATCGGT	*MRLC2*	Myosin regulatory light chain MRLC2	0	0	0	22	44.0
TGGTGTGTGC	*Hs.684687*	cDNA clone ZD94H12	0	0	0	22	44.0
TCCCTATAAA	*no match*	Undefined	0	0	0	18	36.0
AGGACAGCAA	*MYL6*	Myosin, light chain 6	0	1	0	40	27.0
TCCCTAGCCC	*no match*	Undefined	0	0	1	40	27.0
GCTTATAAAA	*HINT1*	Histidine triad nucleotide binding protein 1	0	0	0	13	26.0
TAAACTGTTT	*RPS14*	Ribosomal protein S14	0	0	0	13	26.0
TATGTGTTTT	*PAPSS2*	3'-phosphoadenos 5'-phosphosulf synth 2	0	0	0	13	26.0
TCCCTTTTAA	*B3GNT5*	UDP-GlcNAc:betaGal beta-1,3-N-acetylglucosaminyltransferase 5	0	0	0	13	26.0
TTGAAAATTA	*ANXA2*	Annexin A2	0	0	0	13	26.0
TTGGAACAAT	*no match*	Undefined	0	0	0	13	26.0
TAGACCCCTT	*GAPDH*	Glyceraldehyde-3-phosph dehydrogenase	1	0	0	36	24.0
AAAGGAGAGA	*NUCB2*	Nucleobindin 2	0	0	0	9	18.0
AAGCCTGTAG	*CAMLG*	Calcium modulating ligand	0	0	0	9	18.0

The numbers shown represent the number of tags detected in the indicated libraries per 200,000 total tags. "Fold" indicates the ratio of tags detected in endometriosis vs normen2 (for elevated tags) or normen2 vs endometriosis (for decreased tags). A count of zero (0) tag indicates absence of detection (out of 200,000 tags), not necessarily complete absence of expression in the corresponding tissue.

In addition, further analysis using the SAGE genie database, allowed the identification of genes that are highly specific for endometriosis. SAGE libraries for 29 normal tissues (see "methods" section for the list) were obtained from the SAGE Genie database and compared to the endometriosis libraries constructed for this report. Tags that are highly specific for endometriosis tissues are shown in Table 3. Only tags expressed at a level of more than 20 tags/200,000 in endometriosis and with more than 75% of their total expression found in this tissue were selected. In addition, tags that were found in more than 8 of the 29 normal tissues were also eliminated from the list for lack of specificity. This set of criteria allowed us to identify 20 genes that are highly specific for endometriosis. Interestingly, *MMP*s are prominently represented, with 6 family members (*MMP1, 2, 3, 10, 11, 14*) present among the top 20 endometriosis-specific genes.

**Table 3:** Endometriosis-specific genes

Tag_Sequence	Neg.	Tags in endom	% in endo	Symbol	Gene name
GAGCCAGGCT	27	1249	96.6	*MMP3*	Matrix metallopeptidase 3
TGTCATCACA	22	320	76.3	*LOXL2*	Lysyl oxidase-like 2
TGCAGTCACT	26	317	88.6	*MMP1*	Matrix metallopeptidase 1
TGCAATAGGT	26	265	90.3	*MMP10*	Matrix metallopeptidase 10
GCCAGGTGGC	22	244	81.8	*MMP2*	Matrix metallopeptidase 2
GTATGGGCCC	24	183	88.7	*CHI3L1*	Chitinase 3-like 1
CCGGGGGAGC	27	158	87.5	*COL1A1*	Collagen, type I, alpha 1
CAGGAGACCC	28	144	98.1	*MMP11*	Matrix metallopeptidase 11
CGGGGCGGGG	28	67	83.1	*KISS1*	KiSS-1 metastasis-suppressor
CCCGCCAGTG	27	59	85.6	*ITGA11*	Integrin, alpha 11
CTAAGTAGAG	23	59	56.7	*undefined*	Undefined
GTGCTCAGTG	28	49	78.2	*PXDN*	Peroxidasin homolog (Drosophila)
GTTTTATGCG	27	48	86.8	*DCBLD1*	Discoidin, CUB, LCCL domain containing 1
ACAGAGGGGC	28	47	96.2	*ABBA-1*	Actin-bundling with BAIAP2 homology
CAGTCAATAT	25	43	74.5	*FLJ11041*	FLJ11041
GTACCGGGGA	27	36	80.5	*MMP14*	Matrix metallopeptidase 14
GCTAGACGCG	28	29	86.4	*FOXL2*	Forkhead box L2
CTGTGGTTAC	26	24	77.7	*SLC39A13*	Solute carrier family 39, member 13
CACACAAACA	27	23	75.0	*TWF1*	Twinfilin, actin-binding protein, homolog 1
CAGGCCAACC	28	20	91.6	*RSU1*	Ras suppressor protein 1

"Neg." indicates the number of libraries without detectable expression of the corresponding tags (out of 29 total libraries). "Tag in endom" and "% in endo" indicate the number of tags in endometriosis (per 200,000 total tags), and the percentage of these tags found in endometriosis compared to all the tissues, respectively.

### Validation of SAGE data by immunohistochemistry of selectedcandidates

**Figure 2:**
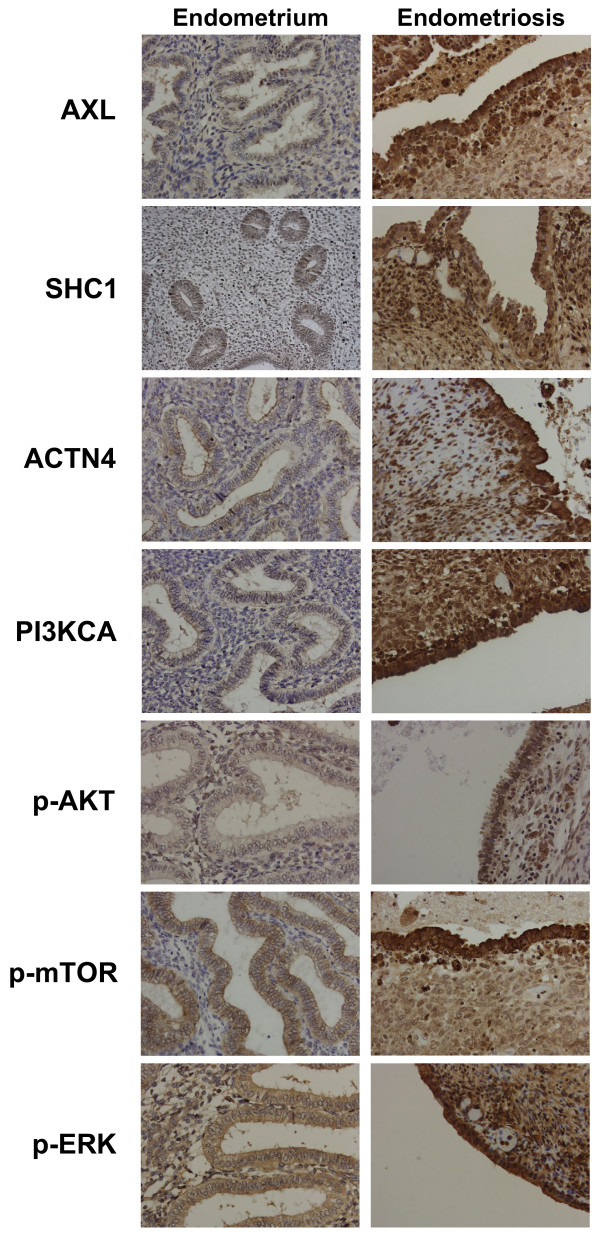
**Immunohistochemical studies of candidates**. Representative immunohistochemical staining of normal endometrium and endometriosis tissues for the indicated proteins (AXL, SHC1, ACTN4, PI3KCA, p-AKT, p-mTOR, and p-ERK) are shown. The candidates shown exhibit increased expression of both epithelial and stromal cells of the endometriotic tissues compared to normal endometrial tissues.

AXL, SHC1, and ACTN4, all shown to be overexpressed in endometriotic epithelial cells by SAGE, were selected for IHC validation. IHC analysis demonstrated that these genes were significantly overexpressed at the protein level in ovarian endometriotic tissues in comparison to normal endometrium for both epithelial and stromal cells (Fig. 2, Table 4). Among the normal endometrium samples, weak to moderate staining of these proteins was observed in those from the proliferative phase, but weak staining was observed in the secretory phase. On the other hand, moderate to strong staining was consistently seen in ovarian endometriosis regardless the menstrual phase (Table 4). Although information about the menstrual phase of three ovarian endometriosis samples was not available, data from the other samples demonstrated that the staining intensity of these proteins in endometriosis was consistently positive regardless of the menstrual phase. Staining of these proteins in normal endometrium was weak and only positive during the proliferative phase.

**Table 4:** IHC staining summary of candidate proteins.

Protein	Cells	Localization	Endometriosis (n = 8)	Endometrium (n = 5)	*P *value
**AXL**	Epithelial cells	Nucleus	1.4 ± 0.74	0.0	0.00061
		Cytoplasm	2.5 ± 0.53	1.2 ± 1.1	0.027
	
	Stromal cells	Nucleus	1.3 ± 0.71	0.0	0.00087
		Cytoplasm	1.5 ± 0.53	0.0	0.000048

**SHC1**	Epithelial cells	Nucleus	1.3 ± 0.46	0.80 ± 0.84	0.16
		Cytoplasm	2.0 ± 0.53	1.2 ± 0.55	0.043
	
	Stromal cells	Nucleus	1.4 ± 0.74	0.20 ± 0.45	0.0023
		Cytoplasm	1.5 ± 0.76	0.40 ± 0.55	0.0059

**ACTN4**	Epithelial cells	Nucleus	1.3 ± 0.71	0.60 ± 0.89	0.11
		Cytoplasm	2.9 ± 0.35	1.6 ± 0.55	0.0017
	
	Stromal cells	Nucleus	1.1 ± 0.99	0.20 ± 0.45	0.022
		Cytoplasm	2.1 ± 0.99	0.40 ± 0.55	0.0022

**PIK3CA**	Epithelial cells	Nucleus	1.9 ± 0.64	0.40 ± 0.55	0.00069
		Cytoplasm	2.5 ± 0.53	1.4 ± 0.55	0.0034
	
	Stromal cells	Nucleus	1.6 ± 0.52	0.20 ± 0.45	0.00021
		Cytoplasm	1.5 ± 0.53	0.20 ± 0.45	0.00042

**p-AKT**	Epithelial cells	Nucleus	1.4 ± 0.74	0.0	0.00061
		Cytoplasm	2.1 ± 0.45	0.80 ± 0.45	0.00058
	
	Stromal cells	Nucleus	1.4 ± 0.74	0.0	0.00061
		Cytoplasm	1.6 ± 0.74	0.0	0.00023

**p-mTOR**	Epithelial cells	Nucleus	1.8 ± 0.46	0.60 ± 0.55	0.0026
		Cytoplasm	2.5 ± 0.53	1.6 ± 0.55	0.0092
	
	Stromal cells	Nucleus	1.4 ± 0.52	0.20 ± 0.45	0.00081
		Cytoplasm	1.3 ± 0.71	0.0	0.00078

**p-Erk**	Epithelial cells	Nucleus	0.63 ± 0.74	0.0	0.025
		Cytoplasm	2.5 ± 0.76	1.6 ± 0.55	0.016
	
	Stromal cells	Nucleus	0.50 ± 0.53	0.0	0.017
		Cytoplasm	1.0 ± 0.53	0.20 ± 0.45	0.0079

Next, we investigated the possibility that downstream pathways in which AXL and SHC1 are involved, PI3K/Akt and MAPK respectively, may be activated in endometriosis. The immunohistochemical analysis for p-ERK, PIK3CA, p-AKT, and p-mTOR demonstrated that all 4 proteins were significantly overexpressed in ovarian endometriomas lining cells compared with normal endometrium (Fig. 2, Table 4). These findings strongly suggest that both MAPK and PI3K/AKT pathways are constitutively activated in ovarian endometriosis.

## Discussion

In this report, we have used SAGE to determine gene expression patterns of ovarian endometriosis and to identify genes differentially expressed in this disease. While other endometriosis gene expression profiling studies have been published, our study is unique for two main reasons. First, the use of SAGE allows an unbiased view of gene expression and is not limited to the genes present on an array. Moreover, it provides an absolute quantitation of gene expression levels, which can then be compared to results from other studies, or other tissues present in public databases. Second, we have put high a high emphasis on isolating pure epithelial endometriotic cells, thus ensuring that the gene expression profiles are representative of the cell type of interest as opposed to representing a mixture of multiple cell types. The endometriosis SAGE libraries obtained here are of extremely high quality and are comprised of approximately 80,000 tags, allowing the detection of genes expressed at relatively low levels. While the complexity of the normal endometrium library was lower, possibly due to the relative difficulty in isolating epithelial endometrial cells in high number, the library was still quite useful with approximately 20,000 tags. It is important to note the relatively low, although typical, number of samples studied by SAGE for this study. The number of samples for SAGE analysis is typically smaller than that used for microarray for various reasons related to the complexity and cost of constructing SAGE libraries [28,29]. On the other hand, the accuracy and reproducibility of the data has been shown to be extremely high [29], and the low number of primary samples studied by SAGE is typically complemented by validation on additional samples using independent techniques.

The issue of cell purity is crucial. While some microarray studies have used whole bulk endometriotic tissues as starting material, others have taken advantage of laser capture microdissection (LCM) for endometriotic cell enrichment. However, LCM leads to RNA of insufficient quality and quantity for SAGE analysis. For these reasons, we used short-time cultivated primary epithelial endometriotic and endometrial cells as starting material. While we focused our study on the epithelial component of endometriosis, it is important to note that there are several reports that suggest a role for the endometriotic stroma in the pathogenesis of endometriosis, and the biological aberrations of the endometriotic stromal cells may be also related, at least indirectly, to malignant transformation of endometriosis to ovarian cancers. However, as a first step in our endometriosis SAGE analysis, we chose to focus on the epithelial component of endometriosis.

Endometriosis, an ectopic lesion of endometrium, has been recognized as a precancerous condition for endometrioid and clear cell ovarian carcinomas [12]. Using MDS, we first show that the gene expression patterns exhibited by the endometriosis samples are very similar to each other and to normal endometrium, but fairly different from epithelial ovarian cancer. This suggests that, if endometriosis can indeed progress to ovarian cancer, extensive changes in genes expression and signaling pathways must occur for this progression to occur. However, it is important to note that the ovarian carcinomas present in the SAGE database and used for this comparison are of serous histology and that endometriosis is not believed to commonly arise from this ovarian cancer subtype. It is possible that endometrioid and clear cell carcinomas would be much more similar to endometriosis than ovarian serous carcinoma and previously published evidence would suggest that it might be the case [12]. Clearly, SAGE analysis of clear cell and endometrioid ovarian cancer would be required to resolve this issue. In any case, the data presented here shows that endometriosis tissues are different from serous ovarian carcinoma tissues in terms of overall gene expression, although it certainly remains possible that certain crucial molecular pathways may be shared between these diseases.

Because SAGE data can be compared between studies and laboratories, we decided to use the large SAGE genie database to identify genes specific to endometriosis by looking for those that are overexpressed in this disease, but that tend to be low in most normal human tissues. We believe that these endometriosis-specific genes may represent useful targets for therapy, as they may be targeted with minimal damage to normal tissues. Using strict criteria for the identification of these genes, we found a number of genes whose expression was relatively specific to the endometriotic tissues studied (Table 3). Remarkably, several MMP genes (*MMP1,2,3,10,11,14*) were found overexpressed and specific for endometriosis, possibly providing a biological explanation for the propensity of these cells to invade the surrounding tissues. Consistent with these findings, upregulation of *MMP1, 2, 3 *has previously been reported in endometriosis [38-42]. MMP expression may be the result of the inflammation associated with endometriosis [43]. In any case, these findings suggest that MMP inhibitors may provide another therapeutic strategy in controlling the spread of endometriosis.

Comparison of gene expression between endometriosis and normal endometrium allowed the identification of a large number of differentially expressed genes. Genes identified as differentially expressed by various previous studies exhibit little overlap with each other and with our current study [13-26]. This may be due to a number of reasons, including differences in sample collection or processing and differences in the profiling platforms. For example, here, we have used eutopic endometrium without endometriosis as normal control tissues. Other studies have used eutopic endometrium from patients with endometriosis as controls, although this clearly may not be ideal. In spite of these differences, we do identify genes that have previously been found in other studies. For example, we show Integrin β1 and Dynein to be upregulated in endometriosis and Matsuzaki and colleagues also documented upregulation of those genes in eutopic endometrium of secretory menstrual phase with deep endometriosis [17]. We also demonstrate upregulation of SHC1 and activation of its downstream pathway, MAPK pathway (discussed below). Activation of the MAPK pathway in endometriosis has also been suggested following the observation that PDGFR, PDGF, and RAF1, are highly elevated in this disease [18]. Furthermore, upregulation of SOS has been demonstrated in eutopic endometrium of late-secretory phase with deep endometriosis [17].

It is well accepted that cell motility and invasion are involved in the pathogenesis of endometriosis, and these function may be related to the oncogenic potential of endometriosis. The present SAGE study reveals that some genes related to cell motility and invasion are indeed deregulated. ACTN4 and Dynein are upregulated, whereas MYL6, ACTG1 and MRLC2 are downregulated (Table 2). Several MMPs such as MMP1, MMP2, MMP 3, MMP10, MMP 11, and MMP 14 are identified as endometriosis-specific genes (Table 3). Furthermore, the proto-oncogenes AXL, SHC1, and JUND are elevated in endometriosis (Table 2).

**Figure 3:**
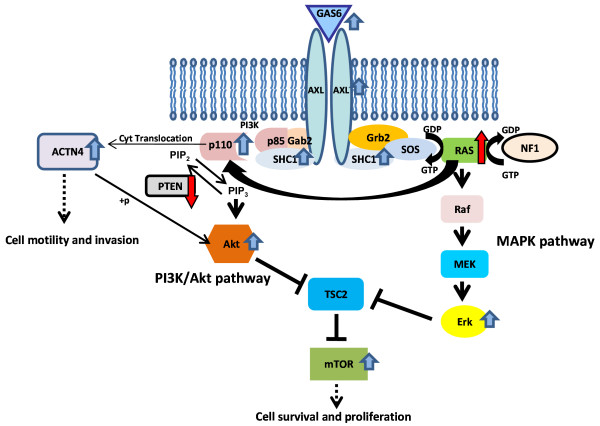
**Schematic representation of molecular pathways possibly involved in the pathogenesis of endometriosis**. The overexpression of SHC1, ACTN4, and AXL observed in the endometriotic samples may lead to the activation of the PI3K and MAPK signaling pathways in human endometriosis. The purple arrows indicate the genes which were shown to be overexpressed in human endometriosis by the SAGE and IHC of the present study. In addition, the red arrows indicate the genes which are activated or inactivated in the mouse model of endometriosis. PIK3CA encodes p110 alpha catalytic subunit of PI3K.

Of particular interest among the differentially expressed genes are those that are involved in well-known signaling pathways that could be inhibited using available drugs. AXL, a member of Tyro3/AXL/Mer (TAM) receptor tyrosine kinase (RTK) family, was shown to be upregulated in endometriosis by SAGE and IHC in the present study. GAS6, a ligand of TAM family, was also found to be upregulated in two of three endometriotic SAGE libraries compared with eutopic endometrium. Overexpression of both AXL and GAS6 in ovarian endometriosis has previously been demonstrated using RT-PCR analysis and IHC [44]. Co-overexpression of AXL with Gas6 has also been reported in several types of tumors including ovarian cancer [45], and may therefore have an important role in uncontrolled cell growth/proliferation. Because the PI3K-Akt signaling pathway is a well known downstream effector of the GAS6/AXL system, we investigated some of the critical components of the PI3K-Akt pathway (PIK3CA, p-Akt, and p-mTOR) in endometriotic tissues using IHC. Similar to what we observed for AXL, the PI3K-Akt signaling pathway was either inactive or weakly active in eutopic endometrium, whereas it was consistently activated in endometriosis. Therefore, it is possible that overexpression of AXL and GAS6 is a main cause for the activation of the PI3K-Akt pathway in endometriosis. As the PI3K-Akt pathway is involved in cell survival, proliferation, and migration, its activation through GAS6/AXL may be a common central biological event during the pathogenesis of endometriosis. Indeed, it was recently shown that activation of K-Ras or inactivation of Pten in mouse ovarian surface epithelial cells can lead to endometriosis-like lesions in the ovary [46]. Since both K-ras and Pten are involved in the PI3K-Akt pathway, alteration of either of these genes could theoretically activates this signaling pathway (Fig. 3).

Similar to what we observed for AXL, SHC1 was found consistently elevated in the studied endometriotic samples. The transforming activity of SHC1 has been established [47], and its overexpression has been reported in neoplasias such as breast, gastric and hepatocelullar carcinomas [48-50]. SHC1 is an adaptor protein that mediates signal transduction in the MAPK pathway, and that can also interact with the p85 regulatory subunit of PI3K. SHC1 could therefore contribute the activation of MAPK and/or the activation of the PI3K-Akt pathway in these tumors. In the current study, we report elevated levels of p-ERK in endometriosis, suggesting activation of the MAPK pathway. Interestingly, this pathway was also shown to be activated in ovarian endometrioid adenocarcinoma derived from endometriosis in a mouse model [46]. The MAPK pathway is therefore constitutively activated in both human endometriosis and in the mouse endometriosis model, and the overexpression of SHC1 is an intriguing mechanism for the activation of this pathway in human endometriosis (Fig. 3). Although, the comparison of gene expression profiles between endometriosis and normal endometrium was done for the proliferative phase, AXL, SHC1, and ACTN4 (discussed below) were also overexpressed during secretory phase in ovarian endometriosis. Continual overexpression of these genes may stimulate cell growth without hormonal stimulation. It is worth noting that these results were obtained through analysis of stage III and IV endometriosis samples. It will be important to extend these findings to early stage endometriosis in order to determine whether these genes may also be useful as early detection markers or therapeutic targets for the prevention of endometriosis development.

ACTN4, which we find strongly expressed in the endometriotic samples, is another candidate gene possibly involved in the pathogenesis of endometriosis. ACTN4 is a non-muscular type of alpha-actinin, a family of actin-bundling protein. Enhanced cytoplasmic expression of ACTN4 is related to increased cell motility and invasion [51] and ACTN4 has recently been implicated in breast, lung, colorectal and ovarian cancers [52-54]. Interestingly, enhanced cytoplasmic expression of ACTN4 is observed in about 60% of endometrioid and clear cell ovarian cancers [54], both of which have been suggested to possibly arise, at least in some cases, from endometriosis. Furthermore, since PI3K can control nuclear translocation of ACTN4, activation of the PI3K-Akt pathway in endometriosis may facilitate enhanced cytoplasmic expression of ACTN4, and may contribute the cell motility and invasion observed in endometriosis.

As discussed above, either oncogenic activation of K-ras or biallelic losses of Pten of ovarian surface epithelium causes endometriosis-like-lesions in the mouse ovary [46]. However, in human endometriosis, no KRAS mutations have been found [55], and alterations of PTEN have been found in only 20% of the cases [8]. As overexpression of AXL and SHC1 theoretically activates both PI3K-Akt and MAPK pathways, dysregulation of these genes in human endometriosis may correspond to oncogenic activation of K-ras or biallelic losses of Pten in the mouse model of the disease. Receptor Tyrosine Kinases (RTKs) are known to potentially activate both the PI3K/Akt and MAPK pathways. However, except for a report showing an increase in c-Met (RTK) and HGF (ligand) in endometriosis [56], there has been little evidence that RTK are overexpressed or activated in this disease.

## Conclusion

Our data show that AXL and SHC1 are differentially expressed in endometriosis implicating the PI3K and MAPK pathways in this disease. AXL may represent a promising new therapeutic target for endometriosis. For example, the use of an AXL-extracellular domain or an AXL dominant-negative receptor mutant for endometriosis treatment may be advantageous compared to conventional therapies such as GnRHa and Danazol. AXL, as well as the other endometriosis-related genes identified in this study, may therefore represent exciting new candidates for the detection and therapy of this disease.

## Competing interests

The authors declare that they have no competing interests.

## Authors' contributions

HH and PJM conceived and designed the study. HH performed SAGE and immunohistochemistry experiments and interpreted the immunohistochemistry results. FFB and DDI provided the clinical specimens used for the SAGE analysis. JG prepared the RNA from the FbEM-1 cell line for SAGE analysis. HH and PJM wrote the manuscript. All authors provided suggestions and inputs on the various drafts of the manuscript. All authors read and approved the final manuscript.
